# Association Between Dynamic Change of QT Interval and Long-Term Cardiovascular Outcomes: A Prospective Cohort Study

**DOI:** 10.3389/fcvm.2021.756213

**Published:** 2021-11-30

**Authors:** Min Ye, Jing-Wei Zhang, Jia Liu, Ming Zhang, Feng-Juan Yao, Yun-Jiu Cheng

**Affiliations:** ^1^Department of Cardiology, The First Affiliated Hospital of Sun Yat-sen University, Guangzhou, China; ^2^Key Laboratory of Assisted Circulation, National Health Commission (NHC), Guangzhou, China; ^3^Department of Medical Ultrasonics, The First Affiliated Hospital of Sun Yat-sen University, Guangzhou, China; ^4^Department of Cardiology, Beijing Anzhen Hospital, Capital Medical University, Beijing, China

**Keywords:** QT interval, sudden cardiac death, coronary heart disease, cardiovascular death, all-cause death

## Abstract

**Background:** The prolongation or shortening of heart rate-corrected QT (QTc) predisposes patients to fatal ventricular arrhythmias and sudden cardiac death (SCD), but the association of dynamic change of QTc interval with mortality in the general population remains unclear.

**Methods:** A total of 11,798 middle-aged subjects from the prospective, population-based cohort were included in this analysis. The QTc interval corrected for heart rate was measured on two occasions around 3 years apart in the Atherosclerosis Risk in Communities (ARIC) study. The ΔQTc interval was calculated by evaluating a change in QTc interval from visit 1 to visit 2.

**Results:** After a median follow-up of 19.5 years, the association between the dynamic change of QTc interval and endpoints of death was U-shaped. The multivariate-adjusted hazard ratios (HRs) comparing subjects above the 95th percentile of Framingham–corrected ΔQTc (ΔQTcF) (≥32 ms) with subjects in the middle quintile (0–8 ms) were 2.69 (95% CI, 1.68–4.30) for SCD, 2.51 (1.68–3.74) for coronary heart disease death, 2.10 (1.50–2.94) for cardiovascular death, and 1.30 (1.11–1.55) for death from any cause. The corresponding HRs comparing subjects with a ΔQTcF below the fifth percentile (<-23 ms) with those in the middle quintile were 1.82 (1.09–3.05) for SCD, 1.83 (1.19–2.81) for coronary heart disease death, 2.14 (1.51–2.96) for cardiovascular death, and 1.31 (1.11–1.56) for death from any cause. Less extreme deviations of ΔQTcF were also associated with an increased risk of death. Similar, albeit weaker associations also were observed with ΔQTc corrected with Bazett's formula.

**Conclusions:** A dynamic change of QTc interval is associated with increased mortality risk in the general population, indicating that repeated measurements of the QTc interval may be available to provide additional prognostic information.

## Introduction

The QT interval on the electrocardiogram (ECG) mainly reflects cardiac ventricular repolarization as abnormal prolongation and shortening of the heart rate-corrected QT (QTc) interval are well-established risk markers for fatal ventricular arrhythmias, sudden cardiac death (SCD), and all-cause mortality in high-risk patients and within the general population (1–4). The hereditary short-QT and long-QT syndromes represent the very extremes of the QTc interval. However, even within the reference range, these altered intervals are correlated with increased mortality risk in the general population (5).

The QT interval is a modifiable factor that may change over time in response to the interaction of genes and environmental factors, resting heart rate, serum electrolyte, as well as clinical conditions, and medical treatment (6). Few studies have assessed the associations of dynamic changes in QT interval over time with mortality. A previous study by Niemeijer et al. has shown that subjects with consistently prolonged QTc interval during follow-up have a higher risk of SCD than those with a consistently normal QTc interval (7). In this study, a prolonged QTc interval was defined as an interval above 450 ms in men and above 470 ms in women. Of note, as a considerable overlap of QTc intervals exists between patients with long QT syndrome (LQTS) and short QT syndrome (SQTS) and truly healthy individuals, it is difficult to use a single QTc value to distinguish all cases of pathogenic “long” or “short” from innocuous variants (8). Besides, the risk for SCD and total mortality is considered to depend on the magnitude of QT shortening or prolongation (9). Selecting rigorous cutoff values to define the normal QTc interval range in population-based studies would be inevitable at the expense of missing some actual patients or overdiagnosing healthy controls as abnormal patients. Thus, the approach to track and assess the QTc interval change as continuous data without definite cutoff values provides a logical rationale for determining the precise dose-response relationship between changes in QTc interval and adverse outcomes in the general population.

To address potential limitations of previous studies, we sought to examine whether a dynamic QTc interval change from the preceding visit is of prognostic importance for SCD and mortality due to cardiovascular disease (CVD) or non-CVD disease in the middle-aged general population in the Atherosclerosis Risk in Communities (ARIC) cohort study.

## Methods

### Study Population

The present study used data from the baseline of the ARIC study. The selection criteria and study design of the ARIC study have been described in detail elsewhere (10, 11). Briefly, the ARIC study is a multicenter, biracial, community-based prospective cohort study that enrolled 15,792 men and women (predominantly white and black) from four US communities (Forsyth County, North Carolina; Jackson, Mississippi; the northwest suburbs of Minneapolis, Minnesota; and Washington County, Maryland), aged 45–64 years at baseline (1987–1989, visit 1). Four short-term follow-up examinations took place at 3-year intervals: 1990–1992 (visit 2), 1993–1995 (visit 3), 1996–1998 (visit 4), and 2011–2013 (visit 5). All participants provided written informed consent, and the study protocol was approved by the local institutional review boards of each participating center.

For this study, we evaluated ARIC participants with ECGs obtained 3 years apart: 1987–1989 (baseline, visit 1) and 1990–1992 (visits 2). We excluded the following participants: (1) subjects for whom ECGs were missing or incomplete for either visit; (2) subjects with ventricular conduction abnormalities (e.g., bundle-branch block, external pacemaker, or Wolff-Parkinson-White pattern); (3) subjects with a history of CVD at baseline that was defined as the presence of ECG evidence of MI, a self-reported history of physician-diagnosed MI, coronary artery bypass surgery, coronary angioplasty, hypertrophic cardiomyopathy, heart failure, and stroke. After all exclusions, the final analysis cohorts consisted of 11,798 ARIC study participants.

### QT Interval Measurement

After 5–10 min of rest, a standard 12-lead ECG with 25 mm/s paper speed and 10 mm/mV voltage settings was performed primarily in the supine position at each visit, by experienced research assistants. A 2-min recording from a three-lead (leads V1, II, and V5) rhythm strip was obtained to conduct measurements manually using calipers. The QT interval measurements were determined by the NOVACODE program, as detailed previously (12). The QT interval was measured from the start of the earliest onset of the QRS complex to the end of the T wave. The end of the T wave was defined as the point of maximum downslope of the T wave and returned to the T-P baseline if not followed by a U-wave. In the present study, all QT interval analyses were corrected for heart rate using both the Framingham formula (QTcF = QT + 0.154 × [1–(60/RR)]) and the Bazett's formula (QTcB = QT/RR^1/2^) (QT in milliseconds and RR in seconds) (13, 14). Prolonged QT interval was defined as QTc ≥470 ms for women and QTc ≥450 ms for men (7). Short QT interval was defined as QTc ≤340 ms (15).

### Mortality Outcomes

Participants in the ARIC study were followed up for mortality through December 31, 2014. The method of follow-up has been detailed previously (16). Hospitalizations and deaths were identified through directed participant queries during annual telephone follow-up. Death certificates and local hospital records were reviewed by ARIC staff to detect additional deaths. The cause of death was defined using the International Classification of Diseases, Ninth Revision and International Classification of Diseases, Tenth Revision codes. Primary outcomes for the present study were SCD, coronary heart disease (CHD) death, CVD death, and death from any cause. SCD was defined as a sudden pulseless condition that was fatal (within 24 h) and that was consistent with a ventricular tachyarrhythmia occurring in the absence of a known non-cardiac condition as the proximate cause of the death (17).

### Statistical Analysis

We used descriptive statistics to summarize the baseline characteristics of participants and the utilization of cardiac medications in the study cohort. Continuous variables are presented as means ± SD and categorical variables as percentages.

We used multivariate Cox hazard models to yield hazard ratios (HRs) and 95%CIs for mortality associated with ΔQTc. The ΔQTc interval was calculated by evaluating a change in QTc interval from visit 1 and visit 2 (indicated as ΔQTcF using Framingham formula; ΔQTcB using Bazett's formula). The analysis was adjusted for age, race, sex, hypertension, diabetes mellitus, currently smoking, body mass index, low-density lipoprotein cholesterol, fasting blood glucose, serum electrolytes (potassium, sodium, calcium, and magnesium), resting heart rate, and use of cardiac medications [β-blockers, calcium channel blockers, QT-prolonging medication (Class IA and Class III antiarrhythmic drugs), and digoxin]. To provide detailed analyses of the dose-response relations of QTc interval change with mortality, we used two alternative methods. First, we examined the relationships of QTc interval change at each cutoff and mortality. In this analysis, we categorized the change of QTc interval into seven groups with cutoffs at the 5th, 20th, 40th, 60th, 80th, and 95th percentiles of the QTc interval change distribution, and used the middle category as the reference group (subjects between the 40th and 60th QTc interval change percentiles, corresponding to the middle quintile). Second, we analyzed the association between hazard and the dynamic change of QTc interval non-parametrically using restricted cubic splines. Five knots were chosen for the analysis to provide a smooth yet flexible description of the dose-response relationship (18). In spline analyses, we used the 50th percentile/the middle quintile of the QTc interval change distribution as the reference value (median). Also, we conducted stratified analyses in men and women separately. Regarding that there may be a potential impact of QT-prolonging medication on QTc interval change during follow-up, we performed a sensitivity analysis of mortality associated with a change of QTc interval with patients taking QT-prolonging medication excluded.

All analyses were performed using commercially available software (STATA, version 15.0; Research Triangle Institute, Research Triangle Park, North Carolina).

## Results

The baseline characteristics of the study group are presented in Table 1. The mean age of the cohort at visit 1 was 54.1 years, 5,332 (45.4%) were men, and 9,357 (79.3%) were Caucasian. A total of 3,768 (31.9%) of the population at baseline had hypertension. With respect to cardiac medications, 632 (5.4%) were receiving β-blocker therapy; 371 (3.1%) calcium-channel blockers; 65 (0.6%) QT-prolonging medication; and 144 (1.2%) were receiving digoxin. The mean duration was 423.2 ms for the QTcF interval and 428.4 ms for the QTcB interval.

**Table 1:** Clinical characteristics of the study population.

**Characteristics**	**Overall (−150 to 163 ms) (*n* = 11,798)**	**Percentiles of** **ΔQTcF and associated interval limits**							***P*-value**
		** <5th (< -23 ms) (*n* = 471)**	**5th−20th (−23 to −8 ms) (*n* = 1,888)**	**20th−40th (−8 to 0 ms) (*n* = 2,360)**	**40th−60th (0–8 ms) (*n* = 2,360)**	**60th−80th (8–16 ms) (*n* = 2,360)**	**80th−95th (16–32 ms) (*n* = 1,887)**	**≥95th (≥32 ms) (*n* = 472)**	
Age (years)	54.1 (5.7)	54.9 (5.8)	54.2 (5.7)	54.0 (5.7)	54.0 (5.7)	54.1 (5.8)	54.1 (5.6)	54.7 (5.6)	0.02
Male sex (%)	5,332(45.2)	196 (41.6)	829 (43.9)	1,063 (45.0)	1,114 (47.2)	1,080 (45.8)	848 (44.9)	202 (42.8)	0.17
White (%)	9,357 (79.3)	308 (65.4)	1,369 (72.5)	1,863 (78.9)	1,905 (80.7)	1,970 (83.5)	1,564 (82.9)	378 (80.1)	<0.001
**Risk factors for vascular events (%)**									
Hypertension	3,763 (31.9)	215 (45.7)	691 (36.6)	717 (30.4)	679 (28.8)	703 (29.8)	573 (30.4)	190 (40.3)	<0.001
Diabetes mellitus	955 (8.1)	53 (11.3)	178 (9.4)	179 (7.6)	179 (7.6)	175 (7.4)	139 (7.4)	52 (11.0)	0.002
Currently smoking	2,680 (22.7)	98 (20.8)	426 (22.6)	556 (23.6)	530 (22.5)	507 (21.5)	446 (23.6)	117 (24.8)	0.41
Body mass index, mean (SD)	27.5 (5.2)	28.9 (5.7)	28.0 (5.4)	27.4 (5.2)	27.3 (5.1)	27.4 (5.1)	27.3 (5.2)	28.2 (5.4)	<0.001
**Laboratory values, mean (SD)**									
**Cholesterol, mean (SD), mmol/L**									
Total cholesterol	4.9 (1.9)	5.1 (1.6)	4.9 (1.9)	4.9 (1.9)	5.0 (1.9)	4.9 (1.9)	4.9 (1.9)	4.8 (1.9)	0.27
Low-density lipoprotein	3.1 (1.4)	3.2 (1.3)	3.0 (1.4)	3.1 (1.4)	3.1 (1.4)	3.1 (1.4)	3.1 (1.4)	3.0 (1.4)	0.26
High-density lipoprotein	1.1 (0.6)	1.2 (0.5)	1.1 (0.6)	1.1 (0.5)	1.2 (0.6)	1.1 (0.6)	1.2 (0.6)	1.1 (0.6)	0.22
Triglycerides	1.4 (1.1)	1.4 (1.0)	1.4 (1.1)	1.4 (1.1)	1.4 (1.1)	1.4 (1.0)	1.4 (1.0)	1.4 (1.0)	0.64
Fasting blood glucose, mean (SD), mmol/L	5.8 (3.0)	5.8 (2.3)	5.7 (3.1)	5.9 (3.2)	5.7 (2.8)	5.7 (3.0)	5.8 (3.1)	5.6 (2.7)	0.11
**Blood electrolyte**									
Serum potassium (mmol/L)	4.4 (0.5)	4.3 (0.5)	4.4 (0.5)	4.4 (0.5)	4.5 (0.5)	4.5 (0.5)	4.5 (0.5)	4.4 (0.5)	<0.001
Serum sodium (mmol/L)	141.0 (2.4)	141.0 (2.5)	141.0 (2.4)	141.0 (2.3)	141.0 (2.4)	141.0 (2.3)	141.0 (2.4)	141.1 (2.4)	0.80
Serum calcium (mg/dL)	9.8 (0.4)	9.8 (0.5 )	9.8 (0.4)	9.8 (0.4)	9.8 (0.4)	9.8 (0.4)	9.8 (0.4)	9.8 (0.5)	0.27
Serum magnesium (mg/dL)	1.6 (0.2)	1.6 (0.2)	1.6 (0.2)	1.6 (0.2)	1.6 (0.2)	1.6 (0.2)	1.6 (0.2)	1.6 (0.1)	0.08
**Electrocardiographic findings**									
Heart rate, bpm	65.6 (9.9)	67.0 (10.3)	66.3 (9.7)	66.3 (9.9)	65.4 (9.6)	65.2 (10.0)	64.5 (9.9)	64.9 (10.9)	<0.001
QT duration, ms	413.1 (28.0)	432.0 (33.7)	420.2 (27.3)	413.4 (26.9)	411.6 (26.3)	409.8 (26.9)	408.5 (26.9)	407.5 (34.1)	<0.001
QTcF, ms	423.2 (20.9)	445.5 (26.4)	432.2 (20.0)	425.1 (18.9)	421.3 (18.7)	418.8 (18.7)	416.1 (19.1)	415.4 (25.2)	<0.001
QTcB, ms	428.4 (24.2)	452.5 (29.5)	438.3 (23.4)	431.1 (22.4)	426.3 (22.1)	423.5 (22.1)	420.0 (22.4)	419.5 (27.4)	<0.001
Atrial fibrillation (%)	21 (0.2)	2 (0.4)	3 (0.2)	1 (0)	7 (0.3)	3 (0.1)	4 (0.2)	1 (0.2)	0.37
QTc interval prolongation consistency (QTcF)									<0.001
Normal-normal	10,833 (91.7)	370 (79.1)	1,756 (93.0)	2,270 (96.2)	2,269 (96.1)	2,235 (94.7)	1,660 (88.0)	273 (58.1)	
Normal-prolonged	490 (4.2)	0 (0)	0 (0)	0 (0)	39 (1.7)	73 (3.1)	196 (10.4)	182 (38.7)	
Prolonged-normal	199 (1.7)	90 (19.2)	90 (4.8)	19 (0.8)	0 (0)	0 (0)	0 (0)	0 (0)	
Prolonged-prolonged	271 (2.3)	8 (1.7)	42 (2.2)	71 (3.0)	52 (2.2)	52 (2.2)	31 (1.6)	15 (3.2)	
**Cardiac medications**									
β-blockers	632 (5.4)	34 (7.2)	111 (5.9)	122 (5.2)	111 (4.7)	132 (5.6)	91 (4.8)	31 (6.6)	0.17
Calcium channel blockers	371 (3.1)	23 (4.9)	73 (3.9)	63 (2.7)	58 (2.5)	65 (2.8)	64 (3.4)	25 (5.3)	0.001
QT-prolonging medication	65 (0.6)	7 (1.5)	13 (0.7)	10 (0.4)	8 (0.3)	13 (0.6)	4 (0.2)	10 (2.1)	<0.001
Digoxin	144 (1.2)	13 (2.8)	25 (1.3)	23 (1.0)	20 (0.9)	18 (0.8)	25 (1.3)	20 (4.2)	<0.001

*QTcF, Framingham–corrected QT interval; QTcB, Bazett–corrected QT interval*.

For QTc interval changes, compared with those in the middle quintile of ΔQTcF, subjects above the 95th percentile or below the 5th percentile of ΔQTcF have older age, higher body mass index, lower serum potassium level, and were more likely to have a history of hypertension and diabetes mellitus, and to receive treatments with calcium channel blockers, QT-prolonging medication, and digoxin. In addition, as the ΔQTcF increases, there was a decrease in heart rate, QT duration, QTcF, and QTcB (Table 1). After a median follow-up of 19.5 years [interquartile range (IQR) 16.2–19.6], a total of 3,315 subjects (28.1%) died. Of these deaths, 344 (2.9%) were adjudicated as SCD. During follow-up, 505 subjects and 760 subjects experienced confirmed CHD death and CVD death, respectively (Table 2).

**Table 2:** A number of deaths by ΔQTcF and ΔQTcb percentiles.

**Percentile**	**Cutoff, ms**	**Subjects**	**SCD (%)**	**CHD death (%)**	**CVD death (%)**	**Death from any cause (%)**
**ΔQTcF**						
<5th	< -23	471	21 (4.5)	30 (6.4)	55 (11.7)	171 (36.3)
5 to <20th	−23 to −8	1,888	69 (3.7)	97 (5.1)	139 (7.4)	553 (29.3)
20 to <40th	−8 to 0	2,360	49 (2.1)	79 (3.3)	121 (5.1)	647 (27.4)
40 to <60th	0–8	2,360	49 (2.1)	70 (3.0)	112 (4.7)	617 (26.1)
60 to <80th	8–16	2,360	58 (2.5)	87 (3.7)	146 (6.2)	631 (26.7)
80 to <95th	16–32	1,887	71 (3.8)	105 (5.6)	138 (7.3)	534 (28.3)
≥95th	≥32	472	27 (5.7)	37 (7.8)	49 (10.4)	162 (34.3)
Total		11,798	344 (2.9)	505 (4.3)	760 (6.4)	33,15 (28.1)
**ΔQTcB**						
<5th	< -30	471	21 (4.5)	32 (6.8)	52 (11.0)	165 (35.0)
5 to <20th	−30 to −11	1,888	66 (3.5)	90 (4.8)	135 (7.2)	538 (28.5)
20 to <40th	−11 to 0	2,360	59 (2.5)	79 (3.3)	122 (5.2)	637 (27.0)
40 to <60th	0–9	2,360	49 (2.1)	82 (3.5)	125 (5.3)	643 (27.2)
60 to <80th	9–19	2,360	63 (2.7)	85 (3.6)	146 (6.2)	620 (26.3)
80 to <95th	19–38	1,887	61 (3.2)	103 (5.5)	136 (7.2)	541 (28.7)
≥95th	≥38	472	25 (5.3)	34 (7.2)	44 (9.3)	171 (36.2)
Total		11,798	344 (2.9)	505 (4.3)	760 (6.4)	33,15 (28.1)

*SCD, sudden cardiac death; CHD, coronary heart disease; CVD; cardiovascular disease; QTcF, Framingham–corrected QT interval; QTcB, Bazett–corrected QT interval*.

### Mortality by QTc Interval Change

**Figure 1:**
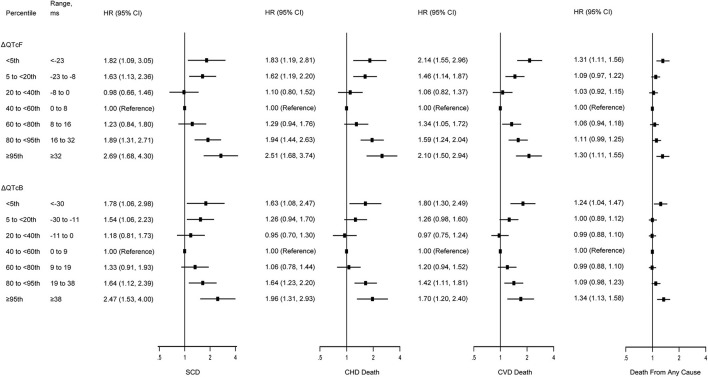
Multivariate-adjusted hazard ratios for SCD, CHD death, CVD death, and death from any cause by risk categories of ΔQTcF and ΔQTcB intervals. SCD, sudden cardiac death; CHD, coronary heart disease; CVD; cardiovascular disease. Models were adjusted for age, race, sex, hypertension, diabetes mellitus, currently smoking, body mass index, low-density lipoprotein cholesterol, fasting blood glucose, serum electrolytes (potassium, sodium, calcium, and magnesium), resting heart rate, and use of cardiac medications (β-blockers, calcium channel blockers, QT-prolonging medication, and digoxin). The vertical dotted lines represent a hazard ratio of 1. The horizontal solid lines represent 95% CI. The QT intervals were corrected for heart rate using either the Framingham formula (QTcF) or Bazett's formula (QTcB).

The associations of dynamic QTc interval change with SCD, CHD death, CVD death, and all-cause mortality are illustrated in Table 2 and Figure 1. Participants with stable QTc intervals during follow-up had the lowest incidences of all outcomes. Both QTc interval prolongation and shortening were associated with higher risks of SCD, CHD death, CVD death, and death from any cause.

**Figure 2:**
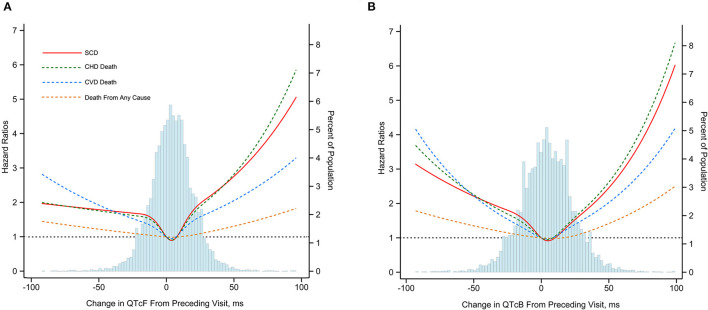
Multivariate-adjusted hazard ratios for SCD, CHD death, CVD death and death from any cause associated with dynamic changes in QTcF **(A)** and QTcB **(B)** Intervals. SCD, sudden cardiac death; CHD, coronary heart disease; CVD; cardiovascular disease. Mortality end points were associated with change of Framingham formula96corrected QT interval (ΔQTcF) and Bazett heart rate96corrected QT interval (ΔQTcB) using restricted cubic splines. The horizontal dotted line indicates a hazard ratio of 1. Adjustment factors are described in Figure 1.

As compared with those in the middle quintile (0–8 ms), subjects above the 95th percentile of ΔQTcF (≥32 ms) experienced increased risks of SCD (HR, 2.69; 95% CI, 1.68–4.30), CHD death (HR, 2.51; 95% CI, 1.68–3.74), CVD death (HR, 2.10; 95% CI, 1.50–2.94), and death from any cause (HR, 1.30; 95% CI, 1.11–1.55) after adjustment for multiple cardiovascular risk factors. In addition, subjects below the 5th percentile of the ΔQTcF (<-23 ms) compared with the middle quintile also had increased risk of SCD (HR, 1.82; 95% CI, 1.09–3.05), CHD death (HR, 1.83; 95% CI, 1.19–2.81), CVD death (HR, 2.14; 95% CI, 1.51–2.96), and death from any cause (HR, 1.31; 95% CI, 1.11–1.56). However, the highest 95th percentile of ΔQTcF seems to confer a higher risk for SCD and CHD death, but not for CVD death and death from any cause compared with the lowest 5th percentile of ΔQTcF. Of note, despite subjects with the extreme 5% highest and lowest ΔQTcF having the highest incidence of death, other categories also were at increased risk compared with the middle quintile (Figure 1). The multivariate-adjusted restricted cubic spline analysis confirmed the U-shaped association obtained from the analysis based on ΔQTcF categories (Figure 2). In this spline-based analysis, subjects with ΔQTcF of 4 ms had the lowest risk of SD and other causes of death, whereas the risk increased for both longer and shorter ΔQTcF.

Similarly, the U-shaped association between dynamic QTc interval change and risk of death was observed when using the ΔQTcB, although the risk magnitude seemed to be slightly decreased (Figure 2). The multivariate-adjusted HRs comparing subjects above the 95th percentile of the ΔQTcB (>38 ms) with subjects in the middle quintile (0–9 ms) were 2.47 (95% CI, 1.53–4.00) for SCD, 1.96 (95% CI, 1.31–2.93) for CHD death, 1.70 (95% CI, 1.20–2.40) for CVD death, and 1.34 (95% CI, 1.13–1.58) for death from any cause. The corresponding HRs comparing subjects below the 5th percentile of the ΔQTcB (< -30 ms) with the middle quintile were 1.78 (95% CI, 1.06–2.98) for SCD, 1.63 (95% CI, 1.08–2.47) for CHD death, 1.80 (95% CI, 1.30–2.49) for CVD death, and 1.24 (95% CI, 1.04–1.47) for death from any cause (Figure 1).

**Figure 3:**
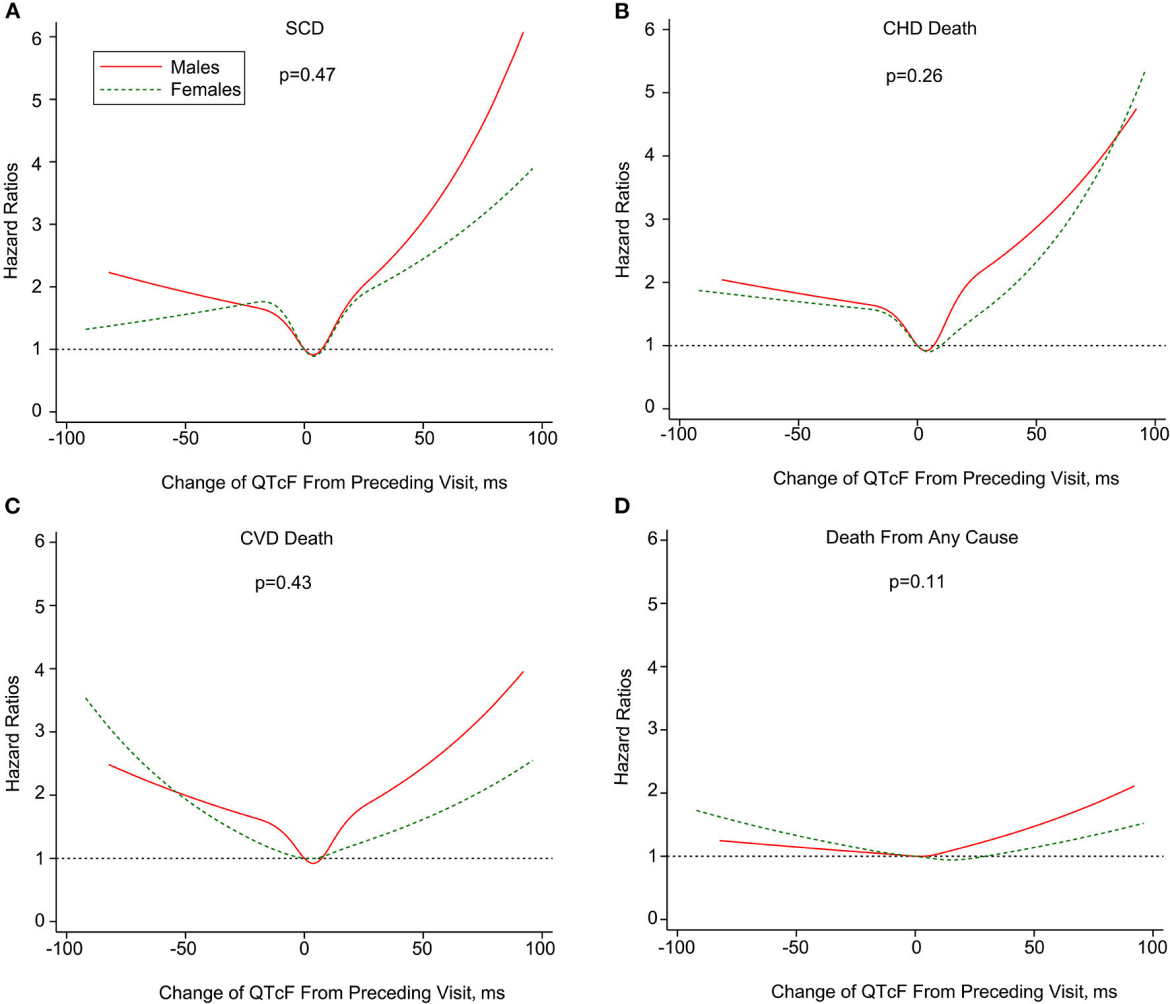
Multivariate-adjusted hazard ratios of SCD **(A)**, CHD death **(B)**, CVD death **(C)** and death from any cause **(D)** associated with dynamic changes in QTcF stratified by sex. SCD, sudden cardiac death; CHD, coronary heart disease; CVD; cardiovascular disease. Mortality end points were associated with change of Framingham formula -corrected QT interval (ΔQTcF) using restricted cubic splines. The horizontal dotted line indicates a hazard ratio of 1. Adjustment factors are described in Figure 1. P values are for interaction.

The U-shaped association of dynamic QT interval change with death endpoints was consistently observed both in men and women. For both genders, QTc interval prolongation and shortening conferred a higher risk of total and cause-specific mortality than relatively stable QTc interval during follow-up. Additionally, the association of ΔQTcF with SCD, CHD death, and CVD death seemed to be more pronounced in men than women, although the difference did not reach statistical significance (Figure 3). Finally, sensitivity analyses by excluding subjects with prolonged or short QT-interval, and those receiving QT-prolonging medication at baseline did not change the results materially (Appendix Table 1 in the Supplementary Material).

### Mortality by QTc Interval Prolongation Consistency

A total of 10,833 (91.8%) of participants with a normal QTcF at visit 1 also had a normal QTcF at visit 2 (normal-normal). However, 490 (4.2%) of the participants with normal QTcF at visit 1 presented with prolonged QTcF at visit 2 (normal-prolonged), 199 (1.7%) participants with prolonged QTcF at visit 1 had normal QTcF at visit 2 (prolonged-normal), and 271 (2.3%) participants had consistent prolonged QTcF for both visits (prolonged-prolonged) (Table 3).

**Table 3:** Multivariate-adjusted hazard ratios for SCD, CHD death, CVD death, and death from any cause by QTc interval prolongation consistency on 2 consecutive ECGs.

**Framingham formula**	**Normal-normal (*n* = 10,833)**	**Normal-prolonged (*n* = 490)**	***P*-value**	**Prolonged-normal (*n* = 199)**	***P*-value**	**Prolonged-prolonged (*n* = 271)**	***P*-value**
SCD	1.00 (reference)	2.29 (1.61–3.27)	<0.001	2.30 (1.40–3.78)	0.001	2.36 (1.52–3.67)	<0.001
CHD death	1.00 (reference)	1.93 (1.42–2.63)	<0.001	1.63 (1.03–2.59)	0.04	2.35 (1.65–3.34)	<0.001
CVD death	1.00 (reference)	1.87 (1.44–2.42)	<0.001	1.90 (1.32–2.73)	0.001	2.63 (1.98–3.50)	<0.001
Death from any cause	1.00 (reference)	1.44 (1.25–1.66)	<0.001	1.32 (1.07–1.63)	0.01	1.71 (1.44–2.03)	<0.001
**Bazett's formula**	**Normal-normal** **(*****n*** **=** **9,976)**	**Normal-prolonged (*****n*** **=** **834)**	***P*** **-value**	**Prolonged-normal (*****n*** **=** **440)**	***P*** **-value**	**Prolonged-prolonged** **(*****n*** **=** **544)**	***P*** **-value**
SCD	1.00 (reference)	1.88 (1.35–2.62)	<0.001	2.08 (1.40–3.09)	<0.001	2.75 (1.98–3.82)	<0.001
CHD death	1.00 (reference)	2.35 (1.83–3.01)	<0.001	1.56 (1.07–2.26)	0.02	2.70 (2.05–3.54)	<0.001
CVD death	1.00 (reference)	1.96 (1.58–2.44)	<0.001	1.70 (1.27–2.29)	<0.001	2.71 (2.17–3.39)	<0.001
Death from any cause	1.00 (reference)	1.44 (1.28–1.62)	<0.001	1.35 (1.15–1.57)	<0.001	1.82 (1.60–2.06)	<0.001

*ECGs, electrocardiograms; SCD, sudden cardiac death; CHD, coronary heart disease; CVD, cardiovascular disease. Models were adjusted for age, race, sex, hypertension, diabetes mellitus, currently smoking, body mass index, low-density lipoprotein cholesterol, fasting blood glucose, serum electrolytes (potassium, sodium, calcium, and magnesium), resting heart rate, and use of cardiac medications (β-blockers, calcium channel blockers, QT-prolonging medication, and digoxin). The QT intervals were corrected for heart rate using either the Framingham formula (QTcF) or Bazett's formula (QTcB). Prolonged QT interval was defined as QTc ≥470 ms for women and QTc ≥450 ms for men*.

**Figure 4:**
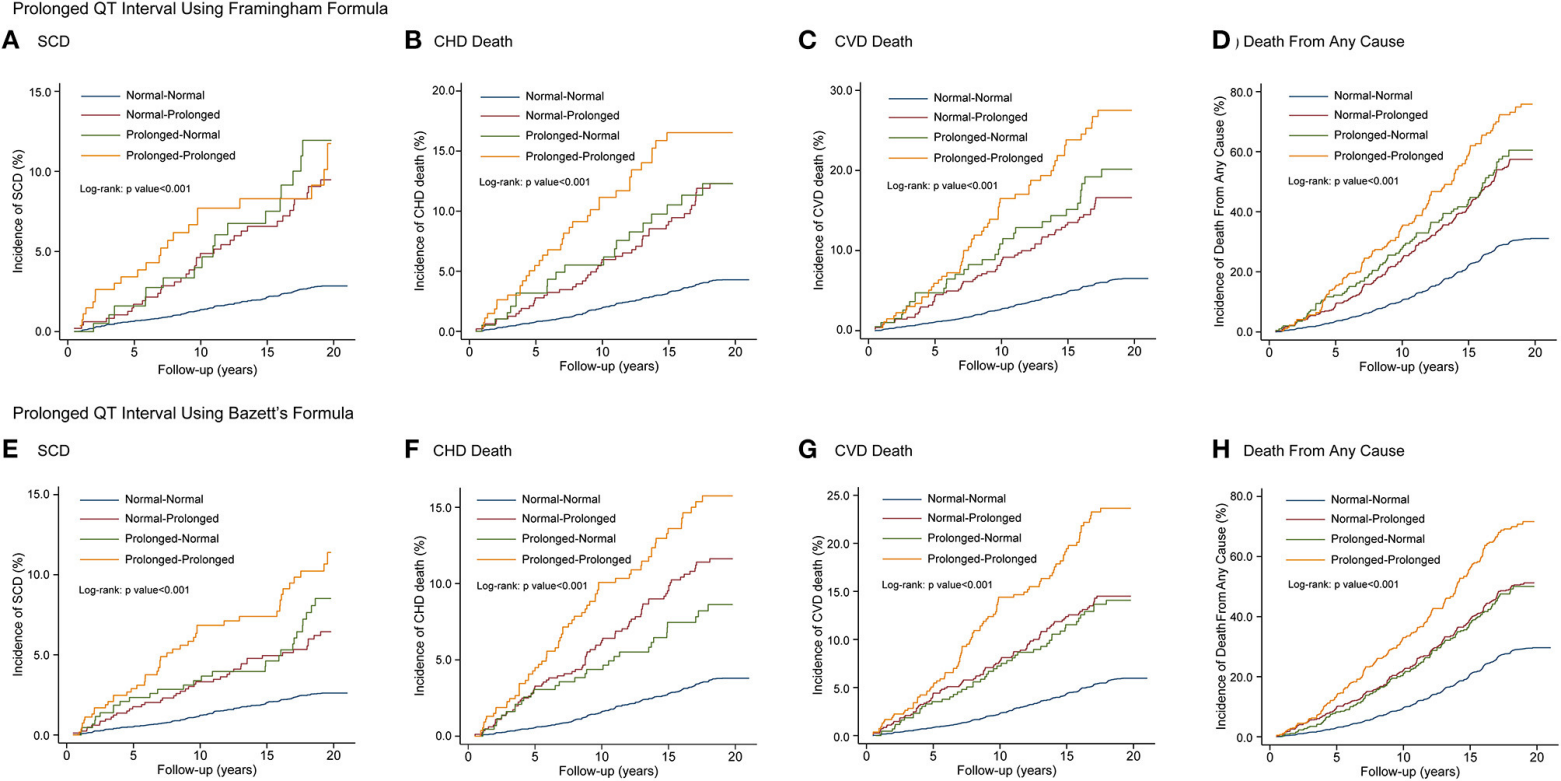
Kaplan-Meier curves of SCD, CHD death, CVD death and death from any cause by QT interval prolongation consistency on 2 consecutive electrocardiograms using QTcF **(A–D)** and QTcB **(E–H)** Intervals. SCD, sudden cardiac death; CHD, coronary heart disease; CVD, cardiovascular disease.

The Kaplan-Meier curves in Figure 4 indicate the association between QTc interval prolongation consistency and risk of death. Compared with those with normal-normal QTcF, subjects with normal-prolonged QTcF experienced an increased risk of SCD (HR, 2.29; 95% CI, 1.61–3.27), CHD death (HR, 1.93; 95% CI, 1.42–2.63), CVD death (HR, 1.87; 95% CI, 1.44–2.42), and death from any cause (HR, 1.44; 95% CI, 1.25–1.66) during the entire observation period. For those with prolonged-normal QTcF, the risks for the four outcomes were significantly increased compared to the normal-normal QTcF group in the multivariable adjusted model. Notably, the risk of SCD (HR, 2.36; 95% CI, 1.52–3.67), CHD death (HR, 2.35; 95% CI, 1.65–3.34), CVD death (HR, 2.63; 95% CI, 1.98–3.50), and death from any cause (HR, 1.71; 95% CI, 1.44–2.03) seem to be even higher in those with prolonged-prolonged QTcF. Similar results were observed for QTcB interval prolongation consistency (Table 3).

The distribution of each event rate across the percentile groups of ΔQTcF in the aforementioned four groups is shown in Appendix Table 2. The incidence rates of adverse outcomes in patients with high ΔQTcF (≥95th) in the prolonged-prolonged group seemed to be higher than those in the normal-prolonged or prolonged-normal group.

## Discussion

In this large prospective cohort study, we observed that both QT interval prolongation and shortening were associated with higher risks of SCD, CHD death, CVD death, and all-cause death compared with a relatively stable QTc interval, with no clear threshold for risk change. Furthermore, there was a U-shaped association between QT interval dynamic change and mortality. The QT interval prolongation seems to be associated with higher risks of SCD and CHD death compared with QT interval shortening. Notably, these associations were not influenced by gender.

Extreme variations in QT interval, which is either “too long” or “too short,” are well-documented risk factors for SCD (15, 19, 20). Individuals with QTc prolongation, indicating prolonged ventricular repolarization, are predisposed to TdP, ventricular fibrillation, and SCD. Besides, subjects with extremely short QT intervals, indicating accelerated repolarization, have also been recently associated with sudden cardiac arrest in individuals with a structurally normal heart. The Rotterdam QT Project followed up a cohort of 6,693 patients who underwent 24-h electrocardiography for 2 years (21). Statistically significantly greater risk for sudden death was noted for subjects with either a shortened (<400 ms) or prolonged (≥440 ms) mean QTc over 24 h, compared with intermediate (400–440 ms) mean QTc values. Recently, Ibrahim et al. reported a strong association between SQTS and incident SCD (22). However, as for the QT interval within a reference range, results have been inconsistent and its associations with mortality risk remained controversial. In the multiethnic study of atherosclerosis (MESA) study with 6,273 U.S. participants aged 45–84 years without prior CVD, Beinart et al. reported that baseline corrected-QT interval was significantly associated with cardiac and vascular events (23). It was demonstrated that the risk of incident heart failure, CVD events, and stroke progressively increased with even a modest QTc prolongation in a dose-response relationship (23). However, as the lack of consistency in demographic characteristics, the measurement method of QT intervals, and the duration of follow-up existed in previous studies, it was difficult to compare the findings and elucidate the dose-response relationships between QT interval variation and mortality risk. Although studies regarding the association between QT-interval duration and mortality risk are extensive, less is known regarding the relationship between change in QT interval from baseline and the risk of death in the general population.

In the present study, we evaluated the association between dynamic changes of QT interval on two consecutive ECGs and mortality risk, setting the relatively stable group as the reference category. We observed U-shaped associations of all-cause and cause-specific mortality with dynamic change of QTc interval in the general population. Another study by Niemeijer et al. examined 3,484 participants with ECGs recorded on two consecutive visits from the Rotterdam Study (7). It was observed that a consistently prolonged QTc interval was associated with a significantly higher risk of SCD, whereas an inconsistently prolonged QTc interval was not significantly associated with increased risk of SCD. In our study, the change of QTc interval was analyzed using restricted quadratic splines with knots at the 5th, 20th, 40th, 60th, 80th, and 95th percentiles to allow a more flexible and non-linear relationship between the dynamic change of QTc interval and mortality risk. Notably, in the present study, we observed that even modest QTc interval shortening and prolongation as compared with the baseline were both associated with a significantly higher risk of mortality. Analyses with categorized variables according to the consistency of two measurements demonstrated consistent findings with those analyzed with the continuous variable of QTc interval change. Compared with those with normal–normal QTc interval, patients with prolonged–prolonged QTc interval experienced the highest risk of mortality, which was in line with those reported by Niemeijer et al. (7). Besides, patients with normal–prolonged or prolonged–normal QTc interval also experienced higher mortality risk than those with normal–normal QTc interval. Further analyses for the distribution of all event rates across the percentile groups of ΔQTcF in the four groups demonstrated that incidence rates of adverse outcomes in patients with high ΔQTcF (≥95th) in the prolonged–prolonged group seemed to be higher than those in normal–prolonged or prolonged–normal group; therefore higher hazards of adverse outcomes were observed in the prolonged–prolonged group as compared with normal–prolonged or prolonged–normal group. These findings indicated that a dynamic change of QTc interval exerts a significantly detrimental effect on outcomes, which was independent of the risk of prolongation of QTc interval. Hence, a more detailed analysis based on the change of serial measurements of QTc interval may provide complementary information on the value of QTc interval for mortality prediction. Further research is required to establish whether changes in the serial measurement of QTc interval are continuously associated with greater mortality risk.

It is well-known that the electrocardiographic QTc interval is approximately normally distributed in the general population (8). Our data further observed that dynamic change of QTc interval in the general population also conformed to a Gaussian normal distribution. Dynamic changes of QTc interval may be caused by multiple factors, including internal (physiologic, pathophysiologic, and genetic) and external (food and drugs) for a given individual. In addition, QTc interval changes markedly during the course of day (24). In the present study, QT-interval was measured based on standard 12-lead resting electrocardiographic recordings of a three-lead (leads V1, II, and V5) rhythm strip. An average QTc was calculated from the analysis of the three-lead rhythm strip, and QTc interval change was assessed between two ECGs recorded under the same conditions (e.g., time of day, posture, and activity), to avoid the interference of the inherent variability of QTc interval over time. To eliminate the possible impact of QT-prolonging medication on ΔQTc, we also performed a sensitivity analysis with exclusion of those taking QT-prolonging medication during follow-up. The results were qualitatively similar to the total cohort. Several mechanisms may explain the association of dynamic changes of QTc interval with an increased risk of cardiovascular and all-cause mortality. Changes in QTc interval were correlated with conditions affecting sympathetic and parasympathetic tone or left ventricular structure, including left ventricular hypertrophy or myocardial infarction (12). In healthy individuals, variation in QTc interval could be a result of disturbance of autonomic nervous system activity (25). Activation of the sympathetic nervous system and inhibition of the parasympathetic system has been recognized as risk factor for coronary atherosclerosis. A high sympathetic tone may increase the propensity for CVD, or the symptoms may occur earlier (25, 26). Furthermore, experimental data showed that QTc interval prolongation was correlated with the occurrence of early after-depolarizations, which has a potential tendency toward cardiac arrhythmias or even progressing to ventricular fibrillation (27, 28). On the other hand, subjects with QTc interval shortening as a continuum might have a different response to pathological and physiological changes such as sinus bradycardia. They have gradually shortened refractory periods both in the atria and ventricles and hence possibly have increased risk for induction of atrial and ventricular arrhythmias (atrial fibrillation/ventricular fibrillation) (29). However, as data regarding the clinical consequences and prognostic significance of moderate QT interval shortening are limited and not conclusive, additional researches are required to determine their peculiar tendency toward spontaneous arrhythmias and their response to therapy. Above all, it is logical to speculate that dynamic change of QTc interval may be a risk marker reflecting the link between ventricular electrical instability and underlying cardiac disease or subsequent serious cardiovascular events, especially in the presence of a high sympathetic tone.

Our findings filled in the gaps about the predictive value of modest prolongation or shortening of QTc interval for mortality risk in the general population. However, our study has several limitations. First, we analyzed the association of mortality risk with the change in QTc interval based on standard 12-lead resting electrocardiography rather than on 24-h Holter monitoring. However, as the universal initial modality in the evaluation of QT interval duration, standard 12-lead ECG appears to be more feasible and practical for serial measurement during follow-up in large studies of general populations, and automatic QT measurements by standard 12-lead resting electrocardiography have been proposed as a reliable mean to accurately measure the QTc interval and have the advantage of greater reproducibility than manual QT measurements (30). Second, atrial fibrillation might bring difficulties in measuring QT interval, which might influence the accuracy of QT interval measurement. However, in the present study, the number of patients with atrial fibrillation was quite small and the proportion of atrial fibrillation among the percentile groups was similar, which would not change the results of the present study. Finally, we assessed the dynamic change of QT interval based on only two measurements. More ECG measurements may be of help to better characterize the association between the dynamic change of QTc interval and mortality risk. However, in this case, it would be crucial to carefully select an appropriate statistical analysis method to unmask the precise dose-response relationship.

In conclusion, we provide evidence that dynamic change of QTc interval was associated with increased risks of SCD, death from CHD, CVD, and all causes in a non-linear manner. Our study showed a U-shaped association between the dynamic change of QTc interval and long-term risk of mortality. A relatively stable QTc interval during follow-up was associated with the lowest risk of deaths. These results indicate that serial measurements of the QTc interval may provide additional prognostic information compared with only a single baseline measurement in the general population. However, future studies are needed to determine the optimal frequency of measurement and unravel the mechanisms underlying the association between the dynamic change of QTc interval and mortality, particularly the relations of moderate changes in QTc interval to mortality.

## Data Availability Statement

The datasets presented in this study can be found in online repositories. The names of the repository/repositories and accession number(s) can be found at: ARIC, https://sites.cscc.unc.edu/aric/.

## Ethics Statement

The study was approved by the institutional review board at all participating institutions and all participants provided written informed consent at enrollment.

## Author Contributions

F-JY and Y-JC: conceptualization and writing—review and editing. MY, JL, and MZ: data curation. MY and J-WZ: formal analysis. MY, J-WZ, and JL: methodology. MZ and F-JY: supervision. MY: writing—original draft. All authors contributed to the article and approved the submitted version.

## Funding

This ARIC study was carried out as a collaborative study supported by National Heart, Lung, and Blood Institute contracts. This study was also financially supported by the grants from National Natural Scientific Foundation of China (81600260), Guangdong Basic and Applied Basic Research Foundation (2021A1515010405), Guangdong Natural Science Foundation (2016A030313210), the project of Guangdong Province Science and Technology Plan (2017A020215174), the Fundamental Research Funds for the Central Universities in Sun Yat-sen University (18ykpy08), the project of Kelin new star of the First Affiliated Hospital of Sun Yat-sen University (Y50186), and the clinical research plan of the Eastern Hospital of the First Affiliated Hospital of Sun Yat-sen University (2019007).

## Conflict of Interest

The authors declare that the research was conducted in the absence of any commercial or financial relationships that could be construed as a potential conflict of interest.

## Publisher's Note

All claims expressed in this article are solely those of the authors and do not necessarily represent those of their affiliated organizations, or those of the publisher, the editors and the reviewers. Any product that may be evaluated in this article, or claim that may be made by its manufacturer, is not guaranteed or endorsed by the publisher.
