# Supplementary material for: Association Between Dynamic Change of QT Interval and Long-Term Cardiovascular Outcomes: A Prospective Cohort Study
Source: Front Cardiovasc Med. 2021 Nov 30;8:756213. doi: 10.3389/fcvm.2021.756213 (PMC8669365; doi:10.3389/fcvm.2021.756213)
Supplement: Supplementary file 1 [file Table_1.pdf]

Appendix Table 1. Multivariate-adjusted hazard ratios for SCD, CHD death, CVD death and death from any cause by risk categories of ΔQTcF and ΔQTcB Intervals.

Percentile	Range, ms	SCD	CHD death	CVD death	Death from any cause
ΔQTcF					
<5th	<-23	1.68 (0.92-3.05)	1.87 (1.14-3.06)	2.08 (1.43-3.04)	1.36 (1.12-1.64)
5 to 20th	-23 to -8	1.47 (1.00-2.17)	1.63 (1.18-2.26)	1.44 (1.11-1.88)	1.05 (0.93-1.19)
20 to 40th	-8 to 0	0.93 (0.62-1.40)	1.10 (0.78-1.54)	1.07 (0.82-1.40)	1.02 (0.91-1.14)
40 to 60th	0 to 8	1.00 (reference)	1.00 (reference)	1.00 (reference)	1.00 (reference)
60 to 80th	8 to 16	1.20 (0.81-1.77)	1.28 (0.92-1.78)	1.34 (1.04-1.74)	1.06 (0.94-1.18)
80 to 95th	16 to 32	1.87 (1.29-2.72)	1.99 (1.45-2.73)	1.62 (1.25-2.09)	1.10 (0.98-1.24)
≥95th	≥32	2.20 (1.31-3.68)	2.46 (1.61-3.74)	1.96 (1.37-2.81)	1.26 (1.05-1.51)
ΔQTcB					
<5th	<-30	1.30 (0.63-2.67)	1.63 (0.92-2.88)	1.83 (1.19-2.82)	1.18 (0.95-1.46)
5 to 20th	-30 to -11	1.50 (0.99-2.25)	1.66 (1.17-2.35)	1.50 (1.13-1.98)	1.07 (0.94-1.21)
20 to 40th	-11 to 0	0.86 (0.55-1.33)	1.10 (0.77-1.58)	1.08 (0.82-1.44)	1.00 (0.89-1.13)
40 to 60th	0 to 9	1.00 (reference)	1.00 (reference)	1.00 (reference)	1.00 (reference)
60 to 80th	9 to 19	1.12 (0.74-1.69)	1.33 (0.94-1.88)	1.38 (1.06-1.81)	1.05 (0.94-1.18)
80 to 95th	19 to 38	1.94 (1.32-2.85)	2.08 (1.49-2.89)	1.69 (1.29-2.21)	1.11 (0.99-1.26)
≥95th	≥38	2.11 (1.23-3.62)	2.52 (1.63-3.91)	1.99 (1.37-2.90)	1.25 (1.04-1.50)

SCD, sudden cardiac death; CHD, coronary heart disease; CVD; cardiovascular disease.Models were adjusted for age,race, sex, hypertension, diabetes mellitus, currently smoking, body mass index, low-density lipoproteincholesterol, fasting blood glucose, serum electrolytes (potassium, sodium, calcium and magnesium), resting heart rate and use of cardiac medications (β-blockers, calcium channel blockers and digoxin). The QT intervals werecorrected for heart rate using either the Framingham formula (QTcF) or Bazett's formula (QTcB).

Appendix Table 2. Number of deaths by QTc interval prolongation consistency and risk categories of ΔQTcF.

QTc interval prolongation consistency (QTcF)	Overall (-150 to 163 ms) (n=11,798)	Percentiles of ΔQTcF and Associated Interval Limits						
		<5th (<-23 ms) (n=471)	5th to 20th (-23 to -8 ms) (n=1,888)	20th to 40th (-8 to 0 ms) (n=2,360)	40th to 60th (0 to 8 ms) (n=2,360)	60th to 80th (8 to 16 ms) (n=2,360)	80th to 95th (16 to 32 ms) (n=1,887)	≥95th (≥32 ms) (n=472)
Normal-normal	10,833	370	1,756	2,270	2,269	2,235	1,660	273
SCD (%)	270 (2.5)	14 (3.8)	56 (3.2)	44 (1.9)	46 (2.0)	47 (2.1)	58 (3.5)	5 (1.8)
CHD Death (%)	405 (3.7)	21 (5.7)	84 (4.8)	71 (3.1)	62 (2.7)	72 (3.2)	85 (5.1)	10 (3.7)
CVD Death (%)	611 (5.6)	38 (10.3)	117 (6.7)	111 (4.9)	97 (4.3)	122 (5.5)	112 (6.8)	14 (5.1)
Death From Any Cause (%)	2,868 (26.5)	129 (34.9)	484 (27.6)	605 (26.7)	574 (25.3)	573 (25.6)	434 (26.1)	69 (25.3)
Normal-prolonged	490	0	0	0	39	73	196	182
SCD (%)	35 (7.1)	0 (0)	0 (0)	0 (0)	1 (2.6)	7 (9.6)	11 (5.6)	16 (8.8)
CHD Death (%)	46 (9.4)	0 (0)	0 (0)	0 (0)	2 (5.1)	7 (9.6)	14 (7.1)	23 (12.6)
CVD Death (%)	64 (13.1)	0 (0)	0 (0)	0 (0)	6 (15.4)	12 (16.4)	19 (9.7)	27 (14.8)
Death From Any Cause (%)	213 (43.5)	0 (0)	0 (0)	0 (0)	19 (48.7)	34 (46.6)	78 (39.8)	82 (45.1)
Prolonged-normal	199	90	90	19	0	0	0	0
SCD (%)	17 (8.5)	6 (6.7)	10 (11.1)	1 (5.3)	0 (0)	0 (0)	0 (0)	0 (0)
CHD Death (%)	19 (9.6)	8 (8.9)	9 (10.0)	2 (10.5)	0 (0)	0 (0)	0 (0)	0 (0)
CVD Death (%)	31 (15.6)	16 (17.8)	13 (14.4)	2 (10.5)	0 (0)	0 (0)	0 (0)	0 (0)
Death From Any Cause (%)	90 (45.2)	37 (41.1)	44 (48.9)	9 (47.4)	0 (0)	0 (0)	0 (0)	0 (0)
Prolonged-prolonged	271	8	42	71	52	52	31	15
SCD (%)	22 (8.1)	1 (12.5)	3 (7.1)	4 (5.6)	2 (3.9)	4 (7.7)	2 (6.5)	6 (40.0)

CHD Death (%)	35 (12.9)	1 (12.5)	4 (9.5)	6 (8.5)	6 (11.5)	8 (15.4)	6 (19.4)	4 (26.7)
CVD Death (%)	54 (19.9)	2 (25.0)	8 (19.1)	8 (11.3)	9 (17.3)	12 (23.1)	8 (25.8)	7 (46.7)
Death From Any Cause (%)	143 (52.8)	5 (62.5)	25 (59.5)	33 (46.5)	24 (46.2)	24 (46.2)	22 (71.0)	10 (66.7)

SCD, sudden cardiac death; CHD, coronary heart disease; CVD; cardiovascular disease.
